# Assessment of histological liver alterations in dogs naturally infected with *Leishmania infantum*

**DOI:** 10.1186/s13071-019-3723-1

**Published:** 2019-10-16

**Authors:** Isadora S. Lima, Manuela S. Solcá, Wagner L. Tafuri, Washington L. C. dos-Santos, Luiz A. R. de Freitas

**Affiliations:** 1 Instituto Gonçalo Moniz, Fundação Oswaldo Cruz, Salvador, BA Brazil; 2 Departamento de Patologia Geral, Instituto de Ciências Biológicas, Universidade Federal de Minas Gerais, Belo Horizonte, MG Brazil

**Keywords:** *Leishmania infantum*, Canine visceral leishmaniasis, Liver histopathology, Pathology, Inflammation, Granuloma

## Abstract

**Background:**

The liver plays a central role in the development of canine visceral leishmaniasis. Studies of natural infection in animals and humans indicate a direct relationship between resolution of infection and the formation and maturation of granulomas in the liver. However, in contrast to other reports in the literature, the present study found no differences in the characteristics of hepatic granulomas that could be related to resistance or susceptibility to *Leishmania*. Here, we describe the hepatic alterations observed in dogs with differing clinical manifestations of visceral leishmaniasis in an endemic area in the state of Bahia, Brazil.

**Methods:**

We examined 148 animals in an endemic area. The animals were clinically examined, and the infection was determined by ELISA, spleen aspirate culture and quantitative PCR. The animals were grouped into asymptomatic or symptomatic based on the number of signs of LV. The histological liver evaluation was performed in a blinded way.

**Results:**

Our results indicated no association between the characteristics of granulomas and clinical presentation. We found an association between the intensity of this inflammatory response and parasite load in the animals’ spleens. It is important to note that while hepatic alterations, such as portal and perivascular inflammation and the presence of larger amounts of granulomas, were linked with higher parasite loads, we found the inverse to be true with respect to intrasinusoidal lymphocytosis, the formation of intrasinusoidal inflammatory cell aggregates and Kupffer cell hypertrophy.

**Conclusions:**

Our findings suggest that the presence of mononuclear inflammatory cells inside the sinusoids is more important than that of organized granulomas in terms of the containment of parasitism by the host. We suggest that the presence of granulomas indicates the failure of a first line of defense mechanism in the control of parasite infection, which could be related to the presence of inflammatory cells and Kupffer cell hypertrophy inside the sinusoids. We further demonstrated that dogs with active *Leishmania* spp. infection present a higher frequency of inflammatory changes in the liver. In addition to being correlated with the severity of clinical manifestation, these hepatic alterations were also associated with changes in hematological and biochemical parameters.

## Background

Visceral leishmaniasis (VL), a zoonosis with a high prevalence and broad distribution throughout the world, is caused by the protozoan *Leishmania infantum* [1, 2]. Dogs are considered the main reservoir of VL as they harbor parasites in the skin, thereby facilitating the infection of sand flies [3, 4]. In addition to the dog’s importance in the epidemiology of VL, the study of canine VL can contribute to the understanding of human disease, since some clinical features, histopathological alterations and disease evolution bear similarities with the human form [5–9].

In humans, different patterns of inflammatory response in the liver have been associated with susceptibility or resistance to VL infection. In susceptible persons, liver alterations include hypertrophy and hyperplasia of Kupffer cells, variable parasitism in these cells, as well as in macrophages in portal tracts, mononuclear cell inflammatory infiltrate in the portal tract and parenchyma, the ballooning of hepatocytes and pericellular fibrosis [10, 11]. Scarce data are available concerning hepatic changes in asymptomatic patients. In the course of an outbreak in Italy, Pampiglione et al. [12, 13] biopsied the livers of five asymptomatic patients with positive DTH for *Leishmania* antigens and observed intralobular granulomas consisting of accumulations of epithelioid macrophages, histiocytes, lymphocytes, plasma cells and rare eosinophils. This same granulomatous inflammatory pattern in the liver has been correlated with resistance in non-susceptible mice [14, 15].

The liver alterations arising from canine VL in naturally infected dogs are akin to those seen in humans. In symptomatic animals, parasitism, inflammatory changes, hyperplasia and hypertrophy of Kupffer cells occur more intensely than in asymptomatic or oligosymptomatic animals [16]. Granulomas of variable size, consisting of macrophages parasitized or not with *Leishmania*, epithelioid cells, small numbers of lymphocytes, plasma cells and rare neutrophils are also described [16–19]. Sánchez et al. [19] reported well-organized granulomas in the livers of asymptomatic dogs that mounted a T-cell memory immune response, in contrast to the disorganized granulomas and intense parasitism of Kupffer cells seen in symptomatic animals. These authors’ findings reinforced the notion that different patterns of inflammatory response correlate with differences in resistance and susceptibility.

An aspect worth considering is that most studies addressing the changes observed in the livers of dogs with visceral leishmaniasis employ experimental models or cases showing extreme poles of the disease, i.e. either asymptomatic or severe clinical manifestations. Although such data are of great importance for the understanding of VL, in fact there exists a wide spectrum of alterations, ranging from cases of subclinical disease with minimal changes to extreme cases that evolve to death, while intermediate cases are crucially important to the general context of this disease. Semi-domiciled and street dogs are largely present in areas endemic for visceral leishmaniasis, such as the area in which our study was conducted. Understanding the inflammatory response and outcome of infection in these animals is essential to the overall understanding of this disease, and can be of great value in the adoption of disease control strategies in these areas, such as immunoprophylaxis.

The present study describes changes in the liver observed in dogs with differing clinical manifestations of visceral leishmaniasis in an endemic area in the State of Bahia, Brazil. Herein we correlate the observed histological hepatic alterations with the severity of clinical manifestations and parasite culture positivity.

## Methods

### Animals

The samples and clinical and laboratory data used in this study were obtained from 148 stray dogs of different breeds and different estimated ages collected from areas around the municipalities of Jequié and Camaçari (Bahia, Brazil), both areas endemic for visceral leishmaniasis, in 2004, 2006, 2010 and 2012. This study was performed in collaboration with the Endemic Diseases Surveillance Programme of the Bahia State Health Secretariat, which is responsible for the surveillance and control of visceral leishmaniasis. All animals were clinically examined by at least two veterinarians. The presence of anti-*Leishmania* antibodies in the sera was determined by ELISA. Dogs with a positive result by ELISA, as well as those who were not claimed by owners, were kept in a kennel for 48 h with free access to food and water. These 148 dogs were then sedated with acepromazine (0.1 mg/kg intravenous (iv), Acepram 1%; Vetnil, Louveira, Brazil) and sodium thiopental (15 mg/kg iv, Thiopentax 1 g; Cristália, São Paulo, Brazil) and euthanized, as mandated per Brazilian Ministry of Health Surveillance Programme protocols, using a saturated solution of potassium chloride (2 ml/kg, iv). Immediately following euthanasia, spleen aspirates were collected for culture and quantitative PCR, and liver and spleen samples were fixed in formalin and embedded in paraffin for morphological studies. The technical details of the anti-*Leishmania* ELISA, splenic culture for *Leishmania* isolation and quantitative PCR have been reported elsewhere [9, 20].

### ELISA

Briefly, 96-well plates were sensitized with crude antigen obtained from *L. infantum*. Plates were washed, blocked with PBS containing 10% skimmed milk, and the serum of each animal was applied at a dilution of 1:400, followed by an anti-dog total IgG peroxidase conjugate (Sigma-Aldrich, San Luis, USA). The enzymatic reaction was developed with tetramethyl benzidine (Sigma-Aldrich). A cut-off was established using serum samples obtained from 48 animals from the municipalities of Porto Alegre (Rio Grande do Sul, Brazil) and Salvador (Bahia, Brazil), both non-endemic areas for visceral leishmaniasis. ELISA results were considered positive when higher than the mean plus three standard deviations (SD) of values obtained from 48 healthy dogs.

### Real-time PCR for the detection of *Leishmania*, *Ehrlichia* and *Babesia* DNA

To detect parasite DNA in frozen spleen samples, DNA was extracted using a DNeasyH Blood and Tissue Kit (Qiagen, Hilden, Germany) in accordance with the manufacturer’s protocols. Once extracted, the quality and concentration of each DNA sample was determined using a digital spectrophotometer (NanoDropH ND-1000; Thermo Fisher Scientific, Waltham, USA). The DNA samples were then adjusted to a concentration of 30 ng/ml, aliquoted and stored at − 20 °C until use. Real-time PCR assays were performed using a previously described amplification procedure [21]. Reactions were performed in a final volume of 25 ml containing 5 ml of the DNA sample diluted to 30 ng/ml in deionized water and 20 ml of PCR mixture. The PCR mixture consisted of 12.5 ml of Universal Mastermix (Applied Biosystems, Carlsbad, CA, USA), 900 nM each of the forward primer LEISH-1 (5′-AAC TTT TCT GGT CCT CCG GGT AG-3′), the reverse primer LEISH-2 (5′-ACC CCC AGT TTC CCG CC-3′) and a fluorogenic probe (5′-AAA AAT GGG TGC AGA AAT-3′), which was synthesized using a FAM reporter molecule attached to the 5′ end and a MGB-NFQ quencher linked to the 3′ end (Applied Biosystems), at a final concentration of 200 nM. A standard curve was generated using serial dilutions of *L. infantum* DNA from 106 to 1021 parasites/ml, with each dilution performed in triplicate. The amplifications were performed in triplicate for each sample and for the negative control using an ABI Prism 5900 sequence detection system (Applied Biosystems). A canine housekeeping gene (*18S* rRNA) was amplified to normalize concentrations of the input sample DNA. Parasite load was expressed as the number of parasites normalized to the established reference amplification value for the *18S* rRNA housekeeping gene in 100 mg of host tissue.

To detect *Ehrlichia canis* DNA in frozen spleen samples, this same protocol was used for DNA extraction, with reactions performed following the protocol described by Bulla et al. [22].

### Clinical data

All animals were subjected to a clinical examination, emphasizing parameters considered indicative of canine visceral leishmaniasis, as defined by Lima et al. [9]. The animals were grouped into two categories according to the recorded clinical signs suggestive of visceral leishmaniasis: asymptomatic (absence of clinical signs) or symptomatic (presence of any clinical signs related to visceral leishmaniasis).

### Biochemistry and hematology

Blood samples for hematological and biochemical analyses were collected from each dog’s cephalic vein under manual restraint. Samples were preserved in EDTA-2Na tubes (Greiner Bio-one, Kremsmünster, Austria) and in blood collection tubes (BD VacutainerH; Becton Dickinson, Franklin Lakes, USA) and examined on the same day. Total red blood cell (RBC) and white blood cell (WBC) counts were obtained using an automated cell counter (Pentra 80 counter; ABX Diagnostics, Montpellier, France). Part of the collected blood was transferred to microhematocrit tubes and centrifuged at 290×*g* for 5 min for hematocrit estimation. Differential blood cell counts were also performed. Serum collected by centrifugation in VacutainerH tubes was used for the following biochemical tests using an enzymatic colorimetric method on an A15 auto-analyzer (BioSystems, Barcelona, Spain): total protein (TP), albumin, aspartate aminotransferase (AST), alanine aminotransferase (ALT), total bilirubin, alkaline phosphatase, urea and creatinine.

### Histological examination

Liver samples were fixed in 10% formaldehyde, dehydrated in graded alcohol, clarified in xylene and embedded in paraffin. Histological sections were cut at a thickness of 4.0 μm and stained by hematoxylin-eosin (H&E) for analysis under conventional light microscopy. Two pathologists (LARF and WLCS) examined all samples. Histological examinations considered the following hepatic alterations: the presence of portal inflammation, central perivenular inflammation (zone 3 of the Rappaport acini), inflammatory cell aggregates within the sinusoids, portal tract granulomas, intralobular granulomas, as well as Kupffer cell hypertrophy, Kupffer cell hyperplasia, sinusoidal congestion and dilatation, intrasinusoidal lymphocytosis, hepatocellular steatosis, ballooning of hepatocytes, hepatocellular necrosis and apoptosis. These aspects were evaluated and classified as absent, mild (score: 1, small aggregates in a small proportion of less than 30% of the examined ×400-magnified microscopic fields), moderate (score: 2, aggregates observed in 30–60% of the examined ×400-magnified microscopic fields) or intense (score: 3, aggregates seen in over 60% of the examined ×400-magnified microscopic fields). A histological score for each animal was calculated by the sum of all observed alterations.

Immunohistochemistry was performed to identify amastigote forms of *Leishmania* in the livers of 20 animals, following a protocol described by Tafuri et al. [23]. Slides were evaluated and the same scoring method described above was applied to grade the presence of amastigote forms of *Leishmania* in the liver.

### Analysis and expression of results

Data were analyzed using STATA Statistics/Data Analysis v.11.0, GraphPad Prism v.5.02 and Microsoft Excel 2013 software. Results are expressed in absolute or relative values. Differences among groups were evaluated by the Kruskal-Wallis test and Dunn’s multiple comparison test to compare between two groups. For comparisons involving proportions, Fisher’s exact test was used. Results were considered significant if *P *< 0.05.

## Results

The clinical and laboratory characteristics of the 148 animals included in this study are presented in Table 1.

**Table 1:** Clinical and laboratory characteristics of the sample

	Frequency	%
*N*	148	100
Sex		
Male	84	57
Female	64	43
Estimated age (years)		
0–2	16/106	15
3–5	73/106	69
≥ 6	17/106	16
Size		
Small	36/146	25
Medium	85/146	58
Large	25/146	17
Clinical category		
Asymptomatic	15	10
Symptomatic	133	90
ELISA		
Negative	27	18
Positive	118	80
Grey zone	3	2
Culture		
Negative	81	55
Positive	67	45
PCR		
Negative	8/135	6
Positive	127/135	94
Leishmanin skin test		
Negative	75/85	88
Positive	10/85	12
Positive in one test	35	24
Positive in all tests	52	35
Co-infections		
Ehrlichiosis	39/57	68
Babesiosis	12/56	21
Positive ELISA results		
Asymptomatic	10	67
Symptomatic	112	84
Positive culture results		
Asymptomatic	3	20
Symptomatic	65	49

*Abbreviation*: N, absolute number. The denominator varies according to the availability of the recorded data

### Histological changes in the liver

**Fig. 1:**
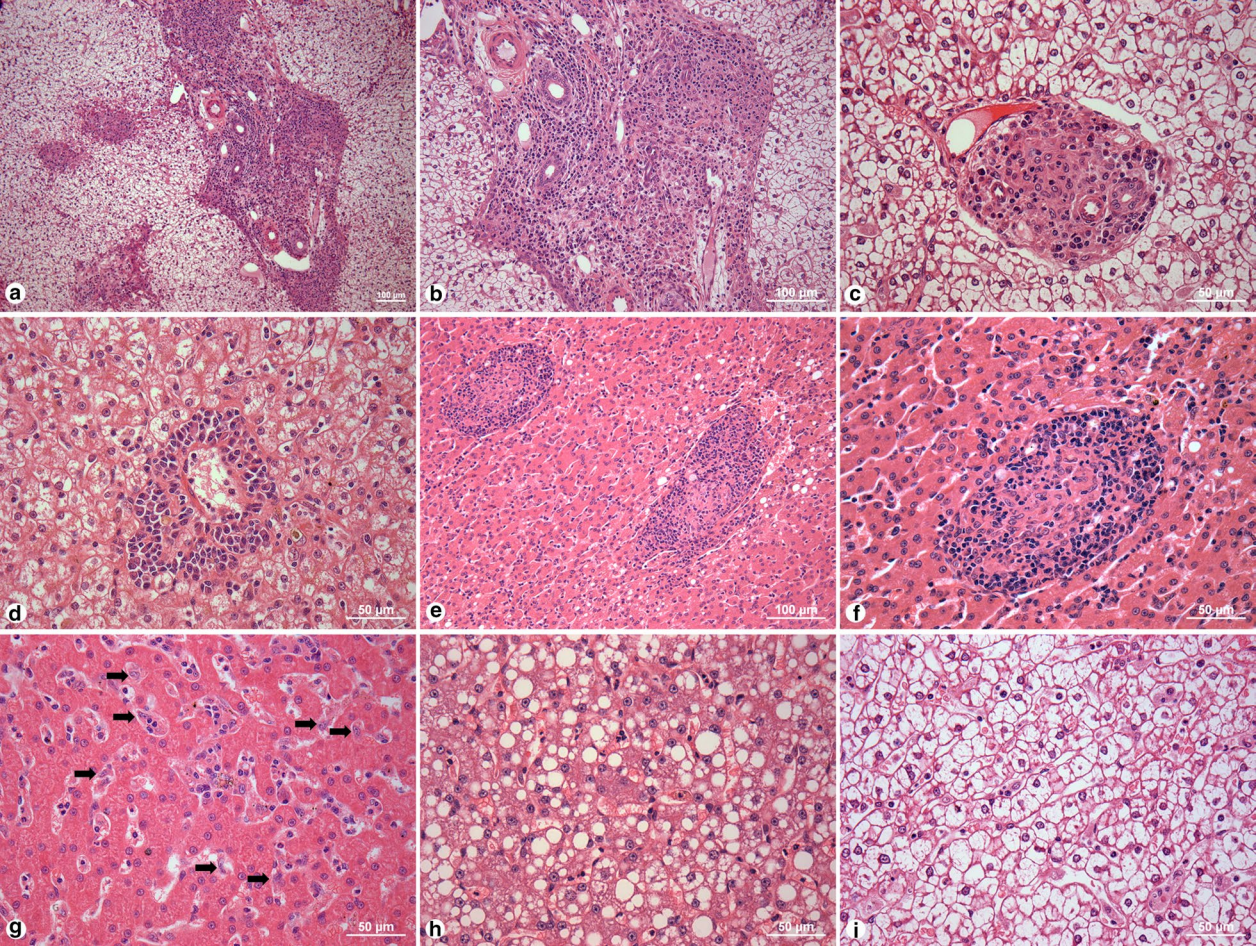
Histological changes in the liver of dogs with *Leishmania* infection. **a**, **b** Portal tract chronic inflammation. **c** Portal tract granuloma. **d** Perivascular inflammation. **e**, **f** Intralobular granuloma. **g** Hyperplasia and hypertrophy of Kupffer cells. **h** Steatosis. **i** Ballooning. *Scale-bars*: **a**, **b**, **e**, 100 µm; **c**, **d**, **f**–**i**, 50 µm

Mild (61%), moderate (28%) or intense (4%) inflammatory infiltrate consisting predominantly of mononuclear cells was present in the portal tracts of 139 (94%) animals (Fig. 1a, b). In 30 (20%) dogs, the portal inflammatory infiltrate had a granulomatous aspect, characterized by the presence of epithelioid cells surrounded by lymphocytes (Fig. 1c). Inflammatory infiltrate consisting of macrophages, lymphocytes and plasma cells was found to be prominent around the centrilobular vein (Fig. 1d). Intralobular granulomas were dispersed throughout the hepatic parenchyma (Fig. 1e, f), varying from loosely organized structures composed of Kupffer cells, which were occasionally parasitized, lymphocytes and plasma cells, to well-organized concentric Kupffer cells surrounded by lymphocytes and rare plasma cells (Fig. 1e, f). Kupffer cell hypertrophy and hyperplasia, as well as steatotic and/or ballooned hepatocytes, were also observed (Fig. 1g–i). The main histological findings observed in the canine liver samples are presented in Fig. 1.

### Correlations between histological hepatic alterations and spleen parasitism

Portal tract inflammation (OR: 16.06, CI: 0.9089–283.8, *P *= 0.0077), portal tract granulomas (OR: 6.727, CI: 2.551–17.74, *P *< 0.0001) and hepatocyte ballooning (OR: 2.614, CI: 1.148– 5.952, *P *= 0.0256) were more frequent in dogs with positive spleen culture than in those without positivity (Fisher’s exact test, Table 2). No statistically significant differences were detected with regard to the frequency of other morphological alterations among the groups.

**Table 2:** Histological alterations in the livers of dogs with and without positive splenic aspirate culture

Histological alteration	With positive splenic aspirate culture (*N *= 68)	Without positive splenic aspirate culture (*N *= 80)
	*n* (%)	*n* (%)
Portal inflammation	67 (99)**	72 (90)
Intrasinusoidal granulomas	44 (65)	43 (54)
Kupffer cell hyperplasia	43 (63)	55 (69)
Kupffer cell hypertrophy	39 (57)	51 (64)
Portal granulomas	24 (35)***	6 (8)
Intrasinusoidal lymphocytosis	51 (75)	64 (80)
Congestion of sinusoids	8 (12)	13 (16)
Dilation of sinusoids	10 (15)	17 (21)
Intrasinusoidal aggregates	38 (56)	47 (59)
Perivascular inflammation	66 (97)	74 (93)
Hepatocyte steatosis	14 (21)	14 (18)
Ballooning of hepatocytes	20 (29)*	11 (14)
Hepatocyte necrosis	10 (15)	15 (19)

* *P *< 0.05, ** *P *< 0.01, *** *P *< 0.005

**Fig. 2:**
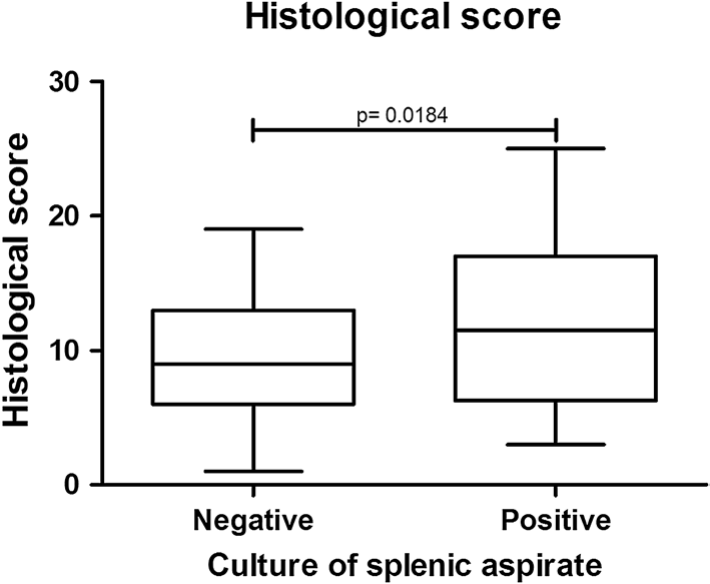
Histological scoring in the livers of dogs with and without positive splenic aspirate culture

**Fig. 3:**
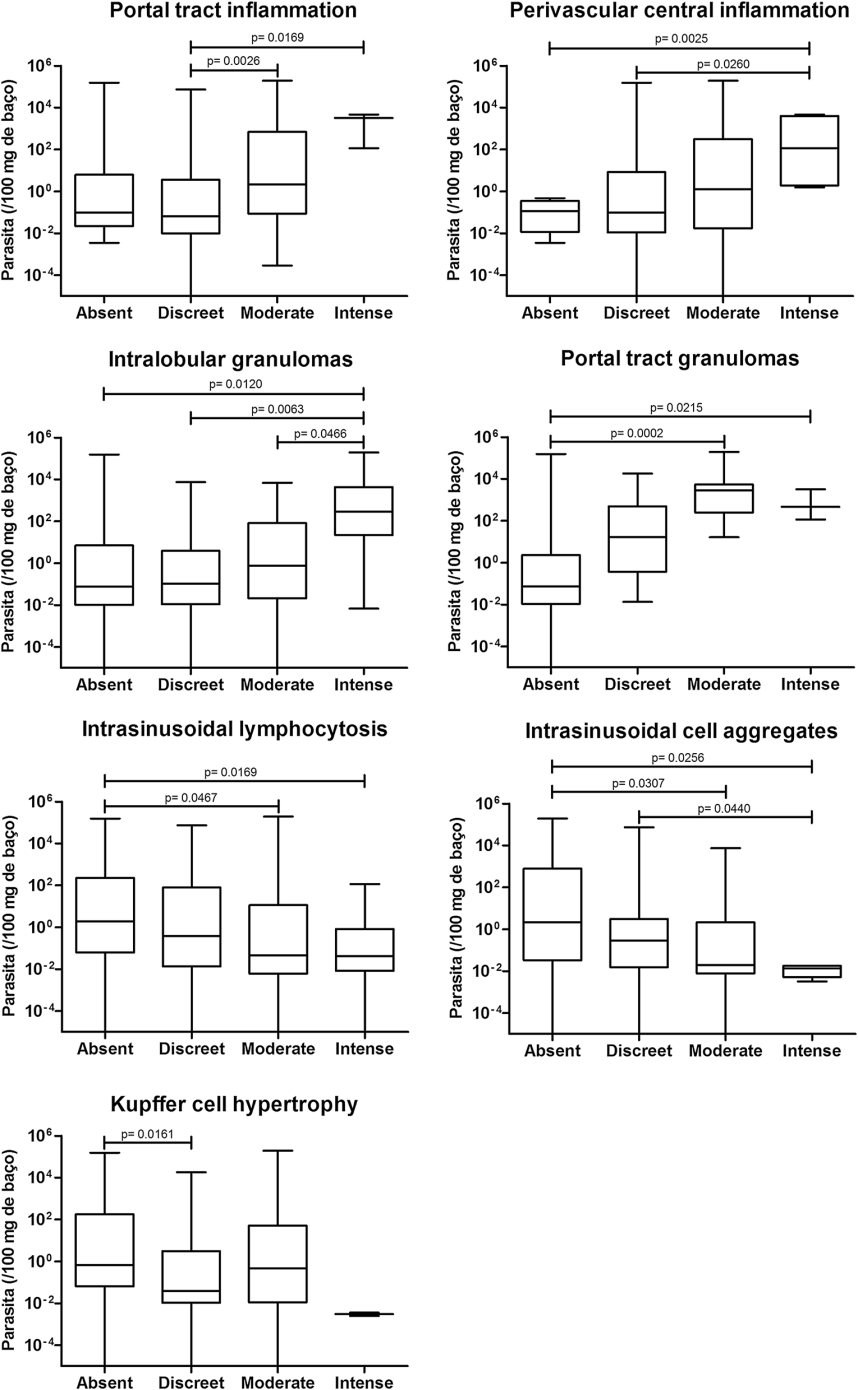
Associations between the intensity of hepatic histological alterations in the liver and splenic parasitism

The mean histological scores were higher in the dogs with positive splenic aspirate culture than in dogs without positivity (Mann-Whitney test, *U *= 2108, *Z *= 2.36, *P *= 0.0184, Fig. 2). Furthermore, splenic parasite burden was higher in animals with intense portal inflammation (Mann-Whitney test, *U *= 21.00, *Z *= 2.39, *P *= 0.0169), intense central perivascular inflammation (Mann-Whitney test, *U *= 0.0, *Z *= 3.02, *P *= 0.0025) and a higher frequency of intralobular (Mann-Whitney test, *U *= 90.00, *Z *= 2.51, *P *= 0.0120) and portal granulomas (Mann-Whitney test, *U *= 33.00, *Z *= 2.30, *P *= 0.0215, Fig. 3). Conversely, higher splenic parasite loads were inversely correlated with intrasinusoidal lymphocytosis (Mann-Whitney test, *U *= 73.00, *Z *= 2.39, *P *= 0.0169), the presence of intrasinusoidal inflammatory cell aggregates (Mann-Whitney test, *U *= 39.00, *Z *= 2.23, *P *= 0.0256) and Kupffer cell hypertrophy (Mann-Whitney test, *U *= 718.5, *Z *= 2.41, *P *= 0.0161, Fig. 3).

### Liver granulomas

Granulomas were classified as disorganized when formed by ill-defined aggregates of epithelioid cells, and well-organized when formed by rounded aggregates consisting of more than 10 epithelioid macrophages. Both types of granulomas showed parasitized macrophages. Lymphocytes and plasma cells were frequently present in well-organized granulomas. Sixteen out of 20 dogs presented granulomas in the liver. Six had organized granulomas and 10 had disorganized granulomas. Among the dogs with organized granulomas, one showed few parasites inside macrophages, two presented intense parasitism, and no amastigotes were observed in the livers of the three remaining animals. In the 10 animals with disorganized granulomas, histological parasitism was scored as mild in three, moderate in two and severe in one dog. No amastigotes were observed in four animals. No statistically significant differences in histological parasitism were detected among the animals with either organized or disorganized granulomas.

### Histological parasitism in the liver

**Fig. 4:**
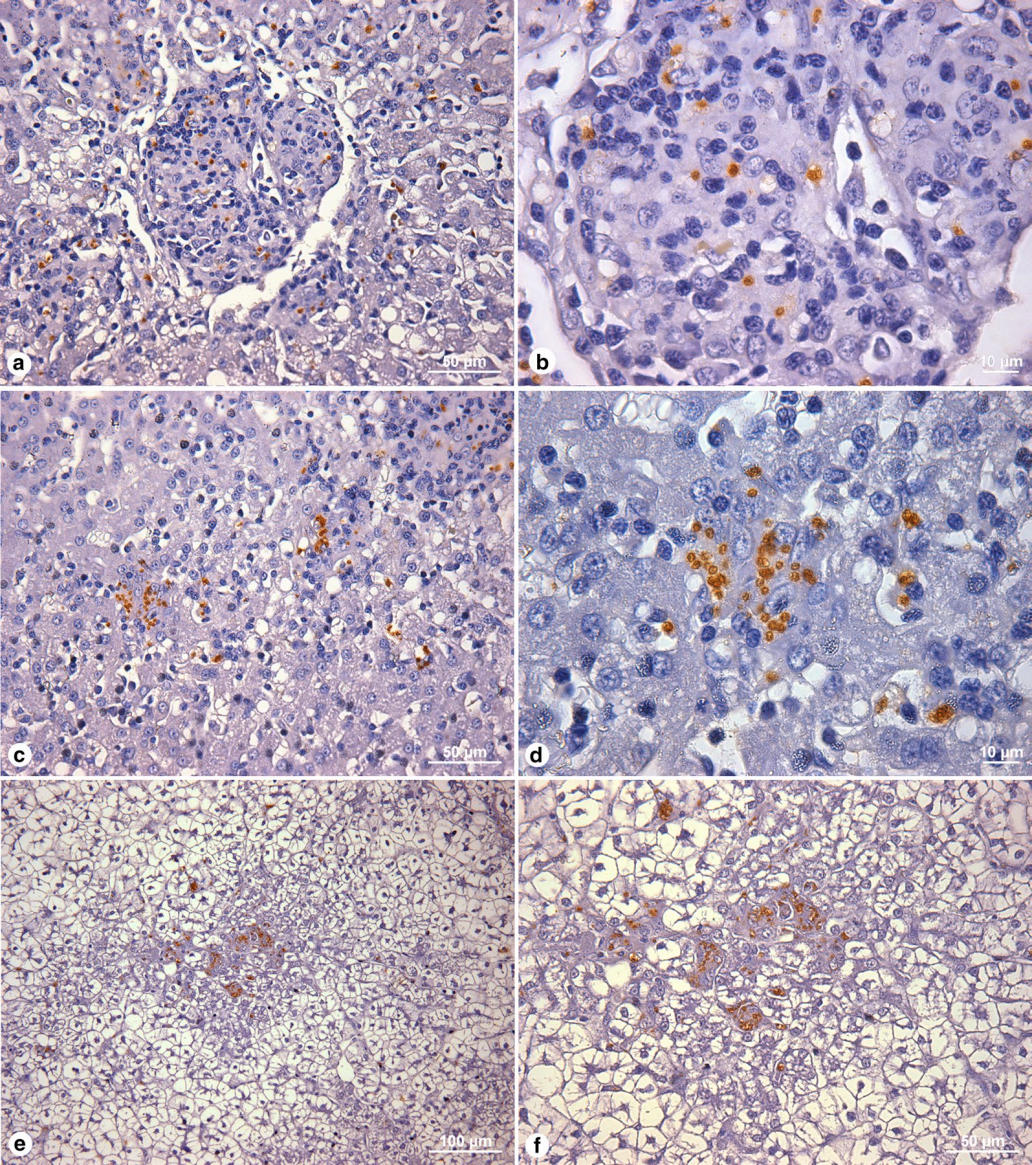
Histological evaluation of the presence of parasites in the liver by immunohistochemistry. **a**, **b** Nodular granulomatous lesion containing amastigotes. **c**, **d** Intracellular amastigotes in Kupffer Cells. **e**, **f** Disorganized granuloma containing amastigotes. *Scale-bars*: **a**, **c**, **f**, 50 µm; **b**, **d**, 10 µm; **e**, 100 µm

Leishmania amastigotes were detected by immunohistochemistry in the livers of 10/20 (50%) animals with different clinical profiles. Histological parasitism was mild in six (30%), moderate in two (10%) and intense in another two (10%, Fig. 4). No statistically significant associations were detected between histological parasitism and the frequency of the other liver histological alterations evaluated.

### Associations between histological alterations and clinical manifestations

As shown in Table 3, animals with symptomatic infection and a positive spleen culture were more likely to present portal inflammation (OR: 3.7, CI: 1.99–6.7), intralobular granulomas (OR: 1.55, CI: 1.1–2.2), portal granulomas (OR: 3.66, CI: 1.8–7.3), perivascular inflammation (OR: 2.76, CI: 1.6–4.8) and hepatocyte ballooning (OR: 1.88, CI: 1.2–2.9) than dogs with asymptomatic infection or those with a negative spleen culture. No statistically significant differences were detected in relation to the other alterations evaluated.

**Table 3:** Histological alterations in the livers of symptomatic dogs with positive *Leishmania* culture *versus* animals with other clinical classifications

Histological alteration	Symptomatic with positive culture (*N *= 65)	Other classifications (*N *= 83)	OR (95% CI)	Adj. OR
	*n* (%)	*n* (%)		
Portal inflammation	64 (98)	75 (90)	3.7 (1.99–6.9)**	3.7**
Intralobular granulomas	42 (65)	45 (54)	1.55 (1.1–2.2)*	1.59*
Kupffer cell hyperplasia	42 (65)	56 (67)	0.93 (0.6–1.4)	1.2
Kupffer cell hypertrophy	38 (58)	52 (63)	0.91 (0.6–1.3)	1.13
Portal granulomas	23 (35)	7 (8)	3.66 (1.8–7.3)**	3.07**
Intrasinusoidal lymphocytosis	50 (77)	65 (78)	1.02 (0.7–1.5)	1.37
Congestion of sinusoids	14 (22)	13 (16)	0.83 (0.4–1.7)	1.23
Dilation of sinusoids	10 (15)	17 (20)	0.82 (0.4–1.5)	0.7
Intrasinusoidal aggregates	38 (58)	47 (57)	1.04 (0.7–1.5)	1.16
Perivascular inflammation	63 (97)	77 (93)	2.76 (1.6–4.8)**	2.9**
Hepatocyte steatosis	14 (22)	14 (17)	1.16 (0.7–1.8)	0.9
Ballooning of hepatocytes	20 (31)	11 (13)	1.88 (1.2–2.9)**	1.39
Hepatocyte necrosis	10 (15)	15 (18)	0.74 (0.4–1.5)	1.05

*Abbreviation*: Adj. OR, odds ratio adjusted re: parasitic burden on the spleen

* *P *< 0.05, ** *P *< 0.01

**Fig. 5:**
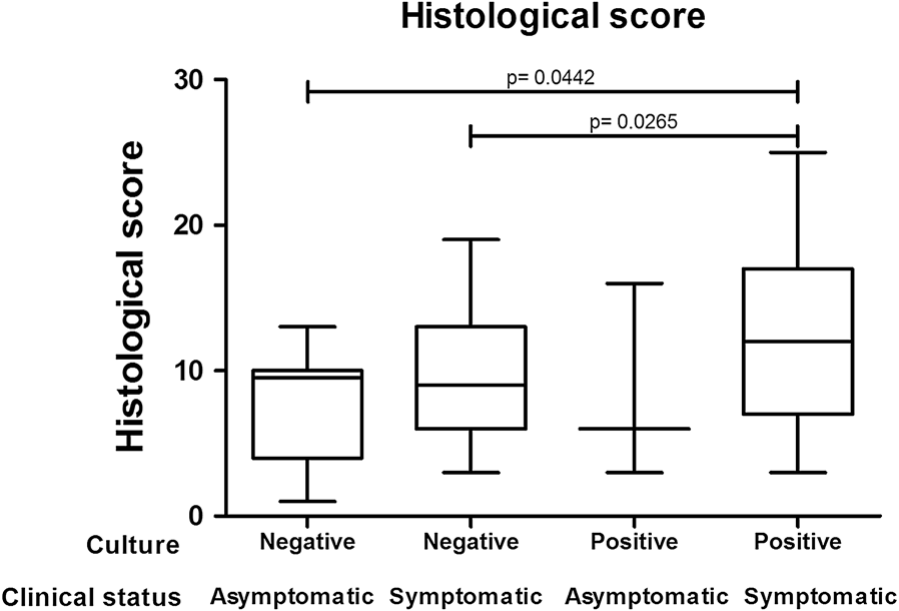
Histological changes in the livers of symptomatic and asymptomatic dogs with and without positive splenic aspirate culture

In addition, animals with active symptomatic infection presented higher histological scores than dogs with a negative spleen culture, regardless of symptomatic (Mann-Whitney test, *U *= 1718, *Z *= 2.22, *P *= 0.265) or asymptomatic status (Mann-Whitney test, *U *= 246.5, *Z *= 2.01, *P *= 0.0442, Fig. 5).

### Associations between hepatic histological alterations and laboratory parameters

RBC counts were lower in animals with intense portal and perivascular inflammation than in animals with mild portal inflammation (Mann-Whitney test, *U *= 6.000, *Z *= 2.54, *P *= 0.0111) or mild perivascular inflammation (Mann-Whitney test, *U *= 8.000, *Z *= 2.81, *P *= 0.0049), as well as animals with moderate portal inflammation (Mann-Whitney test, *U *= 4.000, *Z *= 2.13, *P *= 0.0330) or moderate perivascular inflammation (Mann-Whitney test, *U *= 10.00, *Z *= 2.23, *P *= 0.0257). Hemoglobin concentrations were lower in animals with intense portal and perivascular inflammation than in those with mild portal inflammation (Mann-Whitney test, *U *= 7.500, *Z *= 2.47, *P *= 0.0136) or mild perivascular inflammation (Mann-Whitney test, *U *= 10.50, *Z *= 2.69, *P *= 0.0071), as well as in animals with moderate portal inflammation (Mann-Whitney test, *U *= 3.000, *Z *= 2.25, *P *= 0.0244) or moderate perivascular inflammation (Mann-Whitney test, *U *= 7.000, *Z *= 2.48, *P *= 0.0133). Hematocrit levels were lower in animals with intense portal and perivascular inflammation than in animals with mild portal inflammation (Mann-Whitney test, *U *= 6.000, *Z *= 2.54, *P *= 0.0111) or mild perivascular inflammation (Mann-Whitney test, *U *= 7.000, *Z *= 2.86, *P *= 0.0042), as well as in animals with moderate portal inflammation (Mann-Whitney test, *U *= 4.000, *Z *= 2.13, *P *= 0.0330) or moderate perivascular inflammation (Mann-Whitney test, *U *= 8.000, *Z *= 2.39, *P *= 0.0167, Table 4).

**Table 4:** Associations between histological alterations in the liver and hematological parameters

Histological change	Red blood cells		Hemoglobin		Hematocrit		Leukocytes (×10^3^)	
	Median	p25–p75	Median	p25–p75	Median	p25–p75	Median	p25–p75
Portal inflammation								
Absent	5.78	5.72–5.84 (*n *= 2)	12.5	12.3–12.7 (*n *= 2)	37.4	37.1–37.7 (*n *= 2)	11.4	11.2–11.6 (*n *= 2)
Discreet	5.07*	4.23–5.95 (*n *= 39)	10.6*	9.0–12.8 (*n *= 39)	31.7*	27.0–37.9 (*n *= 39)	14.1	10.3–17.5 (*n *= 39)
Moderate	4.13*	3.88–5.26 (*n *= 15)	9.7*	8.7–11.5 (*n *= 15)	30*	24.5–35.5 (*n *= 15)	13.7	9.0–18.5 (*n *= 15)
Intense	2.73	2.7–3.2 (*n *= 3)	6.6	4.8–7.5 (*n *= 3)	18.2	15.0–21.0 (*n *= 3)	13.2	7.5–16.2 (*n *= 3)
Perivascular inflammation								
Absent	5.72	3.9–5.84 (*n *= 3)	12.3	8.8–12.7 (*n *= 3)	37.1	25.0–37.7 (*n *= 3)	11.6	11.2–15.8 (*n *= 3)
Discreet	5.07*	4.28–5.94 (*n *= 33)	10.6*	9.0–12.8 (*n *= 33)	32.0*	27.6–38.4 (*n *= 33)	13.9	10.2–17.4 (*n *= 33)
Moderate	4.68*	3.96–5.66 (*n *= 19)	10.3*	9.0–11.8 (*n *= 19)	30.4*	25.8–36.7 (*n *= 19)	13.7	9.0–18.5 (*n *= 19)
Intense	2.90	2.7–3.68 (*n *= 4)	6.85	5.25–8.3 (*n *= 4)	18.9	15.8–23.3 (*n *= 4)	14.7	9.52–32.25 (*n *= 4)

*Abbreviations*: p25, 25th percentile; p75, 75th percentile

* *P *< 0.05, ** *P *< 0.01

Serum AST levels were higher in animals with intense portal inflammation than in animals with mild (Mann-Whitney test, *U *= 14.00, *Z *= 2.68, *P *= 0.0074) or moderate portal inflammation (Mann-Whitney test, *U *= 3.000, *Z *= 2.73, *P *= 0.0063, Table 5). Animals with higher numbers (i.e. intense) of intralobular and portal tract granulomas had higher AST levels than animals without intralobular (Mann-Whitney test, *U *= 29.50, *Z *= 2.62, *P *= 0.0087) or portal tract granulomas (Mann-Whitney test, *U *= 7.000, *Z *= 2.64, *P *= 0.0084), and those with mild (Mann-Whitney test, *U *= 26.00, *Z *= 2.49, *P *= 0.0126) or moderate intralobular granulomas (Mann-Whitney test, *U *= 8.000, *Z *= 2.59, *P *= 0.0097). Serum ALT levels were higher in animals presenting intense portal tract granulomas than in animals without this histological alteration (Mann-Whitney test, *U *= 26.00, *Z *= 2.52, *P *= 0.0114, Table 5).

**Table 5:** Associations between histological alterations in the liver and biochemical parameters

Histological change	AST		ALT		Albumin		Globulin		Ratio A/G	
	Median	p25–p75 (*N*)	Median	p25–p75 (*N*)	Median	p25–p75 (*N*)	Median	p25–p75 (*N*)	Median	p25–p75 (*N*)
Portal inflammation										
Absent	30	26–34 (*n *= 2)	32.5	32–33 (*n *= 2)	3	2.7–3.3 (*n *= 2)	6	5.3–6.7 (*n *= 2)	0.5	0.4–0.6 (*n *= 2)
Discreet	33.5**	24.2–51.8 (*n *= 40)	37	27–43 (*n *= 40)	2.85	2.4–3.2 (*n *= 40)	7.45	6–8.8 (*n *= 40)	0.34	0.3–0.5 (*n *= 40)
Moderate	45**	28–54 (*n *= 17)	40	29–65.5 (*n* =17)	2.6	2.1–3.4 (*n *= 17)	7.8	6.5–9 (*n *= 17)	0.35	0.3–0.5 (*n *= 17)
Intense	83	70–112.5 (*n *= 4)	50	22–72.8 (*n *= 4)	2.3	1.7–2.7 (*n *= 3)	6.4	5.4–8.3 (*n *= 3)	0.42	0.2–0.4 (*n *= 3)
Perivascular inflammation										
Absent	34	26–43 (*n *= 3)	32	27–33 (*n *= 3)	3.3*	2.7–3.5 (*n *= 3)	5.3	5.1–6.7 (*n *= 3)	0.62	0.4–0.7 (*n *= 3)
Discreet	34.5	24–54 (*n *= 34)	38	29–46 (*n *= 34)	2.85**	2.4–3.3 (*n *= 34)	7.3	6.2–9 (*n* =34)	0.36	0.3–0.5 (*n *= 34)
Moderate	42	28.5–53 (*n *= 21)	31	27–43 (*n *= 21)	2.7**	2.4–3.4 (*n *= 21)	7.8	6.9–9 (*n *= 21)	0.33	0.3–0.5 (*n *= 21)
Intense	70	48–105 (*n *= 5)	72	30–75 (*n *= 5)	1.8	1.7–2.2 (*n *= 4)	6.5	5.6–7.9 (*n *= 4)	0.28	0.2–0.4 (*n *= 4)
Intrasinusoidal aggregates										
Absent	47	31–58 (*n *= 18)	39.5	30–60 (*n *= 18)	2.7	2.4–3.3 (*n *= 17)	6.7**	5.3–7.9 (*n *= 17)	0.43*	0.3–0.6 (*n *= 17)
Discreet	38	25–53 (*n *= 21)	34	27.5–38.5 (*n *= 21)	3	2.2–3.5 (*n *= 21)	6.7**	5.6–7.6 (*n *= 21)	0.42**	0.3–0.6 (*n *= 21)
Moderate	33.5	27–51 (*n *= 18)	38.5	27–46 (*n *= 18)	2.65	2.4–3.2 (*n *= 18)	8.5	7.4–9.7 (*n *= 18)	0.295	0.3–0.4 (*n *= 18)
Intense	51	21–74 (*n *= 6)	40	18.5–84 (*n *= 6)	2.4	2.1–3 (*n *= 6)	8.2	6.8–10.2 (*n *= 6)	0.265	0.2–0.4 (*n *= 6)
Intralobular granulomas										
Absent	35**	28.5–49.5 (*n *= 25)	34	26–42.5 (*n *= 25)	2.7	2.05–3.2 (*n *= 25)	7.1	6.1–8 (*n *= 25)	0.34	0.3–0.5 (*n *= 25)
Discreet	38*	24–61.5 (*n *= 21)	35	27–43 (*n *= 21)	3	2.4–3.4 (*n *= 21)	7.4	6.4–9.8 (*n *= 21)	0.4	0.3–0.5 (*n *= 21)
Moderate	31.5**	25–48.8 (*n *= 10)	38.5	29.2–44.8 (*n *= 10)	2.6	2.4–3.4 (*n *= 10)	7.9	6.4–9 (*n *= 10)	0.34	0.3–0.5 (*n *= 10)
Intense	75	49–110 (*n *= 7)	72	36–82 (*n *= 7)	3	2.2–3.7 (*n *= 6)	8	5.4–9.5 (*n *= 6)	0.38	0.3–0.55 (*n *= 6)
Portal granulomas										
Absent	34**	25–48 (*n *= 55)	36*	27–43 (*n *= 55)	2.8	2.4–3.3 (*n *= 55)	7.5	6.4–8.8 (*n *= 55)	0.34	0.3–0.5 (*n *= 55)
Discreet	68	51.8–97 (*n *= 4)	40.5	30.5–47.5 (*n *= 4)	3	2.5–3.45 (*n *= 4)	6.9	5.6–7.7 (*n *= 4)	0.44	0,3–0,6 (*n *= 4)
Moderate	68	68–68 (*n *= 1)	20	20–20 (*n *= 1)	2.3	2.3–2.3 (*n *= 1)	5.4	5.4–5.4 (*n *= 1)	0.43	0.4–0.4 (*n *= 1)
Intense	90	75–120 (*n *= 3)	73	72–120 (*n *= 3)	2.15	1.7–2.6 (*n *= 2)	8.75	8.3–9.2 (*n *= 2)	0.24	0.2–0.3 (*n *= 2)

*P < 0.05, **P < 0.01

Serum levels of albumin were lower in animals with intense perivascular inflammation than in animals without perivascular inflammation (*U *= 0.0, *Z *= 1.96, *P *= 0.0498), as well as in those with mild (*U *= 8.000, *Z *= 2.83, *P *= 0.0046) or moderate perivascular inflammation (Mann-Whitney test, *U *= 6.000, *Z *= 2.64, *P *= 0.0083, Table 5). Serum globulin levels were higher in animals with a moderate number of intrasinusoidal aggregates than in animals without (*t *= 2.788, *P *= 0.0087) this alteration, or those with low numbers (i.e. mild) of intrasinusoidal aggregates (*t *= 3.697, *P *= 0.0007, unpaired t-test, Table 5).

Serum triglyceride levels were higher in animals with higher numbers of intralobular granulomas than in animals without (*U *= 11.00, *Z *= 2.72, *P *= 0.0066) this alteration, as well as in those with lower numbers (i.e. mild) of intralobular granulomas (*U *= 8.000, *Z *= 2.72, *P *= 0.0065). Serum cholesterol levels were higher in the animals that presented intense perivascular inflammation than in those with mild (*U *= 2.000, *Z *= 2.67, *P *= 0.0075) or moderate perivascular inflammation (Mann-Whitney test, *U *= 0.0, *Z *= 2.66, *P *= 0.0077, Table 6). No significant differences were found among the groups with respect to the other biochemical or hematological tests evaluated.

**Table 6:** Associations between histological alterations in the liver and biochemical parameters such as triglyceride and cholesterol

Histological change	Triglyceride		Cholesterol	
	Median	p25–p75 (*N*)	Median	p25–p75 (*N*)
Perivascular inflammation				
Absent	28	25–31 (*n *= 2)	159.5	157–162 (*n *= 2)
Discreet	46	31–64 (*n *= 31)	165**	130–233 (*n *= 31)
Moderate	57.5	44.2–81.2 (*n *= 18)	177.5**	157.8–215.8 (*n *= 18)
Intense	82	41–83 (*n *= 3)	332	269–448 (*n *= 3)
Intralobular granulomas				
Absent	44.5**	32.5–66.2 (*n *= 22)	169	153–219.5 (*n *= 22)
Discreet	47.5**	31.8–59.5 (*n *= 18)	167	134.8–202 (*n *= 18)
Moderate	55	41–69.5 (*n *= 9)	175	139–222 (*n *= 9)
Intense	83	72–102 (*n *= 5)	236	130.5–360.5 (*n *= 5)

*Abbreviation*: p25, 25th percentile; p75, 75th percentile

** *P *< 0.01

Our analysis of associations between histological hepatic alterations and PCR for *Erlichia* and *Babesia* indicated that dogs with positivity for *Ehrlichia* presented smaller portal granulomas (*U *= 287.5, *Z *= 2.03, *P *= 0.0423) and a more intense intrasinusoidal lymphocyte infiltrate (Mann-Whitney test*, U *= 218.0, *Z *= 2.43, *P *= 0.0152,) compared to negative animals. No statistical differences were found in relation to the other parameters evaluated.

## Discussion

The present study found an association between a positive splenic culture for *L. infantum* and parasite burden in the spleen in dogs with inflammation in the liver. While hepatic portal and perivascular inflammation, as well as granulomas, were linked to higher parasite loads in the spleen, intrasinusoidal lymphocytosis, intrasinusoidal mononuclear inflammatory cells aggregates and Kupffer cell hypertrophy were associated with lower splenic parasite loads. Additionally, liver inflammation was also correlated with clinical, hematological and biochemical alterations. No significant associations were found between histological parasitism of the liver and the frequency of the other histological hepatic alterations, or granuloma organization.

It is interesting to note that different patterns of inflammatory infiltrate in the liver seem to be associated with different stages of infection, as well as difference in the immune response to *Leishmania* [14]. It has been shown in mice and hamsters that sequential increases and decreases in parasite burden in the liver are followed by a sustained increase in splenic parasitism [14]. Hence, intra-sinusoidal leukocytosis and the presence of leukocyte aggregates in association with a low parasite burden in the spleen may represent an early stage of parasite dissemination in internal organs. Another possible explanation is that this pattern of inflammatory infiltrate in the liver may represent a more effective immune response to infection.

Chronic inflammation has been associated with the persistence of infection [15]. Furthermore, evidence indicates that the organization and cell composition of chronic inflammatory infiltrate may be associated with protection against or permissiveness to the infecting agent [19, 24]. For instance, reports in the literature have described well-organized granulomas in association with resistance to *Leishmania* in experimental models, as well as in human disease [12, 24]. Herein, the frequency and morphological characteristics of granulomas bore no relation to clinical manifestations in the animals evaluated, nor was any association with the control of parasitism observed. In contrast, well-organized granulomas containing many parasites in their interior were observed. Granulomas are functionally dynamic structures that may initially contain parasites, which they may be able to eliminate, or not. Although the formation of granulomas may constitute a step towards infection control, parasites may be able to subvert granulomas to favor survival and growth [24]. Hence, as shown in an experimental model of leishmaniasis [24] as well as in tuberculosis, granulomas in dogs may also be permissive or non-permissive to *Leishmania*, and can either eliminate parasites, or favor their survival [25, 26]. The fact that Kupffer cell hypertrophy was found to be associated with decreased parasitism in the spleen may suggest that granulomas represent a second line of defense after Kupffer cell activation failed to kill parasites. It follows that the presence of granulomas may provide evidence that the first-line attempt at parasite elimination was unsuccessful, as a subsequent chronic inflammatory response is mounted. An improved characterization of cellular phenotype, in addition to the production of cytokines and microbicidal factors by intra-sinusoidal leukocytes, may help to better understand liver inflammation in VL.

We also found that symptomatic dogs with a positive spleen culture had a higher frequency of inflammatory changes in the liver compared to the other groups. In addition, these animals were more likely to have a higher overall clinical score. While similar hepatic alterations have been described in the literature [9, 16, 17, 27–29], differences in clinical classification and the presence of infection may explain some of the discrepancies between our results and those reported by other studies, such as the fact that we did not use uninfected control animals for comparisons. The present study considered liver alterations only in dogs naturally infected by *Leishmania* from an endemic area, which presented the full clinical spectrum of disease.

In addition to clinical signs, the present study also evaluated hematological and biochemical parameters, revealing an association between histological inflammatory changes and laboratory alterations. A previous study reported a positive correlation between the progression of Kupffer cell hyperplasia and hypertrophy and increases in serum globulin and total protein levels, which was found to be negatively correlated with serum albumin concentrations [16]. Regarding the other biochemical and hematological alterations, the relationship between these alterations and a more severe clinical picture in dogs with VL has already been described in the literature. However, previous reports did not evaluate these alterations with respect to histological alterations in the liver, but rather in relation to clinical manifestation, parasite load and the disorganization of splenic white pulp, which differs from the criteria evaluated in the present study [9, 30].

Our analysis of the presence of co-infections found an association between the size of portal tract granulomas, the intensity of intrasinusoidal lymphocytosis and positivity by PCR for *Ehrlichia canis*. The relatively scare studies that reported an association between ehrlichiosis and histological changes in the liver found periportal and perivascular mononuclear inflammatory cell infiltrate, sinusoidal microgranulomas, steatosis and sinusoid congestion [31, 32]. We believe that this warrants further investigation.

## Conclusions

The present findings raise the possibility that dogs exhibit a response profile different from that described in murine models. Our results suggest that the activation of Kupffer cells and increased numbers of lymphocytes in the liver, as well as the formation of aggregates of these cells inside the sinusoids, may be responsible for the elimination of parasites, leading to the containment of infection. In summary, it is possible that granuloma formation may be the result of the failure of the first-line of parasite load and infection control.

## Data Availability

Data supporting the conclusions of this article are provided within the article. The datasets used and/or analyzed are available from the corresponding author upon reasonable request.

## Abbreviations

VL: visceral leishmaniasis
IHC: immunohistochemistry
H&E: hematoxylin and eosin
DTH: delayed type hypersensitivity
OR: odds ratio
CI: confidence interval
ALT: alanine aminotransferase
AST: aspartate aminotransferase
RBC: red blood cell
WBC: white blood cell
TP: total protein
ELISA: enzyme-linked immunosorbent assay
iv: intravenous
PCR: polymerase chain reaction
EDTA-2Na: ethylenediaminetetraacetic acid, disodium salt
p25: 25th percentile
p75: 75th percentile
